# Intra-aortic balloon pump treatment in an adult patient with a Fontan circulation and acute heart failure: a case report

**DOI:** 10.1093/ehjcr/ytae289

**Published:** 2024-06-15

**Authors:** Miriam Sjåstad Langseth, Njord Nordstrand, Gunnar Erikssen

**Affiliations:** Department of Cardiology, Oslo University Hospital, Rikshospitalet, PB 4956 Nydalen, 0424 Oslo, Norway; Department of Cardiology, Oslo University Hospital, Rikshospitalet, PB 4956 Nydalen, 0424 Oslo, Norway; Department of Cardiology, Oslo University Hospital, Rikshospitalet, PB 4956 Nydalen, 0424 Oslo, Norway

**Keywords:** Intra-aortic balloon pump, Adult congenital heart disease, Fontan circulation, Acute heart failure, Case report

## Abstract

**Background:**

There is limited evidence for the use of an intra-aortic balloon pump (IABP) in adult patients with a total cavopulmonary, or Fontan circulation.

**Case summary:**

A patient in his twenties with a Fontan circulation presented with sepsis, pneumonia, and pulmonary oedema. He was born with a hypoplastic left ventricle, atrioventricular septal defect, and hypoplastic aortic arch, and a total cavopulmonary circulation had been established within his first years of life. Standard of care treatment with antibiotics, non-invasive ventilatory support, loop diuretics, and vasopressors was initiated. Due to persistent pulmonary congestion and increasing general fatigue, an IABP was placed via a femoral artery to offload the failing systemic ventricle. Secondary to IABP treatment, mean arterial pressure rose, and vasodilatory nitroprusside could be introduced. Over 4 days of IABP treatment, the patient’s general condition and ventricular systolic function improved significantly.

**Discussion:**

This case suggests that IABP treatment was important in the recovery of our patient with a Fontan circulation, pneumonia, and heart failure. We propose that during IABP treatment, an increase in stroke volume and a reduction in ventricular filling pressure is achieved, thereby increasing the transpulmonary pressure gradient that is central to pulmonary blood flow in Fontan patients. More definitive evidence is necessary to confirm our hypotheses.

**Learning points**

- The Fontan circulation is critically dependent on the transpulmonary gradient generated between the central venous pressure and ventricular filling pressure.
- The haemodynamic effects of intra-aortic balloon pump treatment may be particularly useful in Fontan patients with sepsis-induced cardiomyopathy, due to this dependency on the transpulmonary gradient.

## Introduction

To our knowledge, the evidence for the use of intra-aortic balloon pump (IABP) treatment in adult Fontan patients is limited to three case reports.^1–3^ Even among children with a Fontan circulation, the use of IABP is a rarity, and in such cases mostly used in the post-operative setting shortly after establishment of Fontan circulation.^3–6^ We present an adult Fontan patient with acute pneumonia and heart failure, treated with IABP. Our observations suggest that IABP treatment made an important contribution to recovery.

## Summary figure

**Figure:**
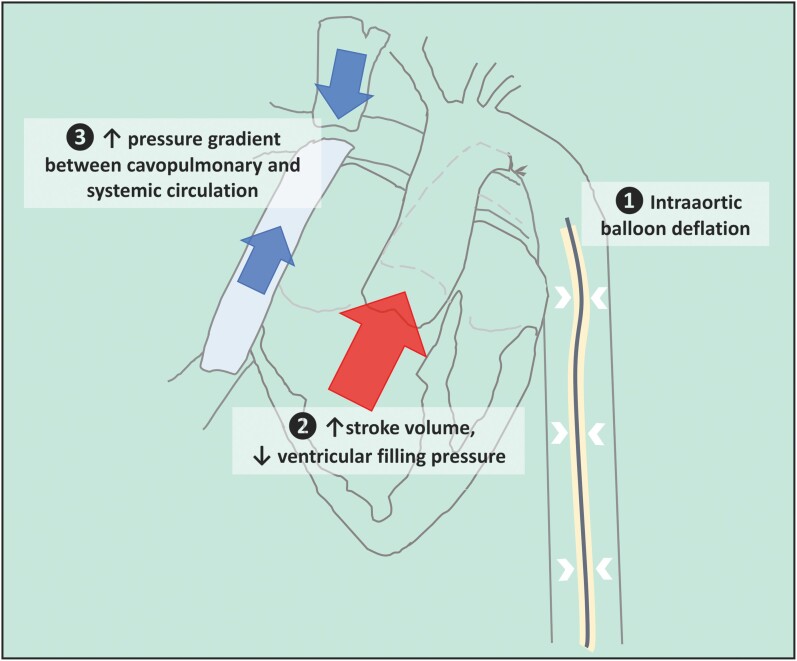


## Case presentation

The patient is a male in his early twenties born with a hypoplastic left ventricle, unbalanced atrioventricular septal defect, subaortic stenosis, aortic arch hypoplasia, and right isomerism with azygous continuation and asplenia. He was born to term weighing 3100 g after an uncomplicated pregnancy. According to standard of care, a Norwood 1 procedure with aortopulmonary shunt and Damus–Kaye–Stansel (DKS) anastomosis was performed on the second day of life. At 7 months, a bidirectional cavopulmonary connection was established, and at 16 months, a total cavopulmonary connection using an extracardiac conduit.

The patient suffered a cerebral haemorrhage after Norwood 1, leaving him with hydrocephalus and cerebral palsy. A ventriculoperitoneal shunt was implanted at 2 months, and revised at age 21 due to shunt failure. Due to paroxysmal atrial fibrillation, the patient was anticoagulated with rivaroxaban. Annual echocardiography showed ventricular ejection fraction (EF) 40%, a mild neo-aortic regurgitation, and a moderate atrioventricular valve insufficiency. Due to asplenia, pneumococcal vaccination had been recommended, but not administered since 2003.

The patient presented to his local hospital with a 12 h history of increasing breathlessness and fatigue. Initial review showed pulse oximetry 76% (habitually >90%), and body temperature 37.9°C. The patient was drowsy, had sinus tachycardia with heart rate 120–130 beats per minute, blood pressure 125/81 mmHg, respiratory rate 28 with intercostal retractions, global pulmonary rales/crackles, and prolonged capillary refill time (4–5 s). Initial blood gas analysis showed type I respiratory failure with metabolic acidosis and partial respiratory compensation. Point-of-care lung ultrasound showed a substantial number of B-lines.

Initial treatment consisted of bronchodilatory nebulizers with oxygen 10 L/min, and 250 mg hydrocortisone and 2 L of crystalloid fluid given intravenously. Broad-spectrum cephalosporin antibiotics were given to treat possible community-acquired pneumonia, and noradrenaline to maintain adequate blood pressure due to possible sepsis (procalcitonin 2.5 µg/L). Non-invasive ventilatory support (CPAP) with FiO2 85% was started due to respiratory deterioration and rising lactate to >4. After initial stabilization, the patient was transferred to the tertiary care centre, (anonymized hospital name for submission), for specialized anaesthetic expertise.

**Figure 1:**
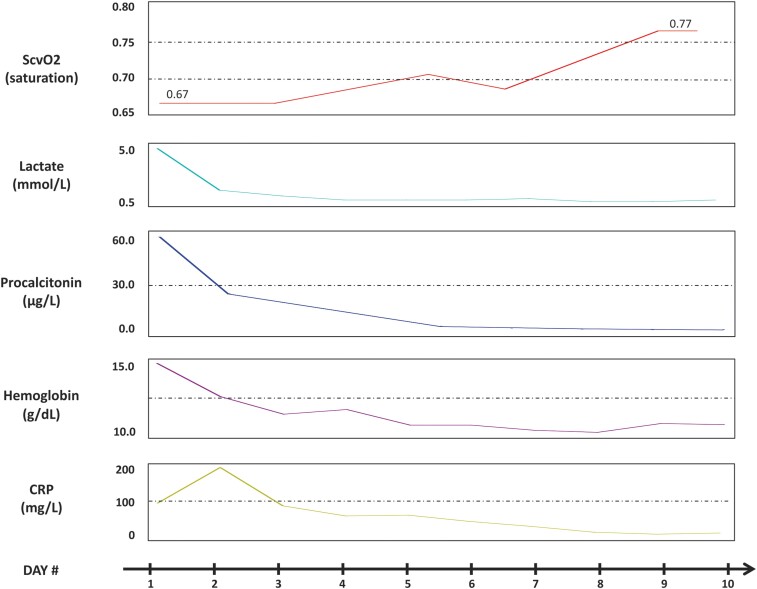
The temporal profiles of selected circulating biomarkers: central venous oxygen saturation (ScvO2), lactate, procalcitonin, haemoglobin, and C-reactive protein (CRP).

**Figure 2:**
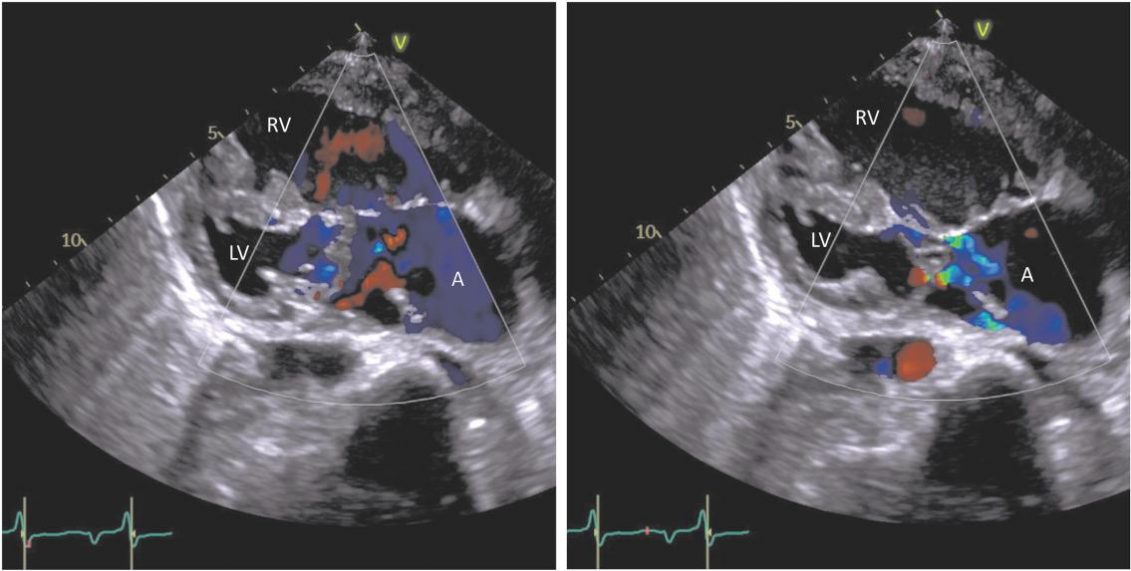
Modified parasternal long-axis view of the systemic right ventricle (RV), hypoplastic left ventricle (LV), atrial complex (A), and atrioventricular valves, demonstrating end-diastolic (left) and end-systolic (right) dimensions and AV-regurgitation on arrival at the tertiary care centre.

**Figure 3:**
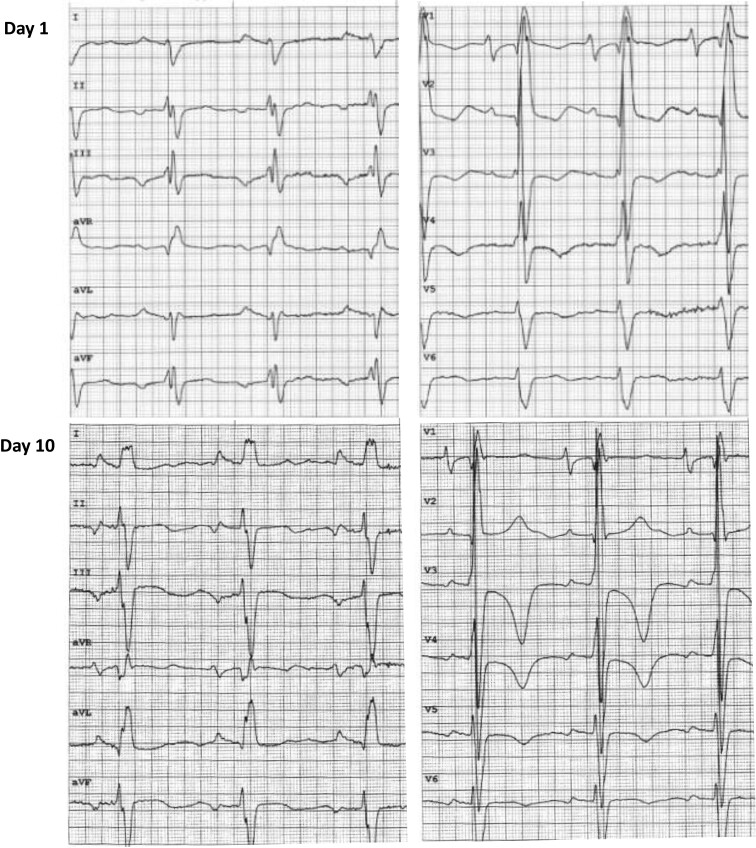
Electrocardiogram (ECG) on Day 1 and Day 10, showing sinus rhythm, ventricular hypertrophy, and delayed intraventricular conduction (QRS duration ∼140 and 120 ms, respectively). ECG paper speed 50 mm/s.

**Figure 4:**
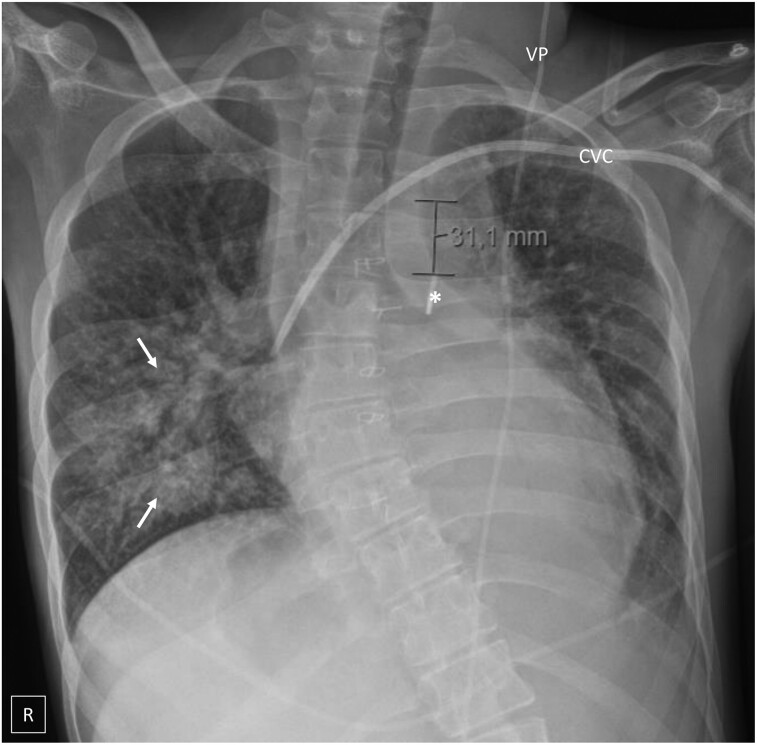
Chest X-ray on Day 3 showing consolidation of the right lower lobe (arrows), generalized alveolar shadowing and upper lobe venous diversion, as well as a central venous catheter (CVC) and ventriculoperitoneal shunt (VP). The X-ray demonstrates intra-aortic balloon pump placement with the radiopaque marker (asterisk) roughly 3 cm distal to the aortic knob, deliberately lower than standard placement due to aortic arch hypoplasia and previous surgical procedures.

The temporal profile of selected circulating biomarkers is shown in *Figure 1*. NT-proBNP was initially > 35 000. Echocardiography showed a severely hypokinetic ventricle with EF 20% (*Figure 2*, Supplementary material online, *Videos S1* and *S2*). An electrocardiogram (ECG) showed sinus tachycardia and delayed intraventricular conduction (QRS duration 140 ms), which complicates interpretation with respect to ischaemia (*Figure 3*). High-sensitive troponin T measured 108 ng/L (URL 14) on Day 1 and fell to 44 on Day 2. Point-of-care Influenzae and COVID-19 viral tests were negative. Urinalysis was positive for pneumococcal antigen, and a nasopharyngeal PCR test revealed coinfection with respiratory syncytial virus. Blood cultures were negative. A chest X-ray showed a right lobar pneumonia and alveolar shadowing consistent with pulmonary oedema (*Figure 4*).

**Figure 5:**
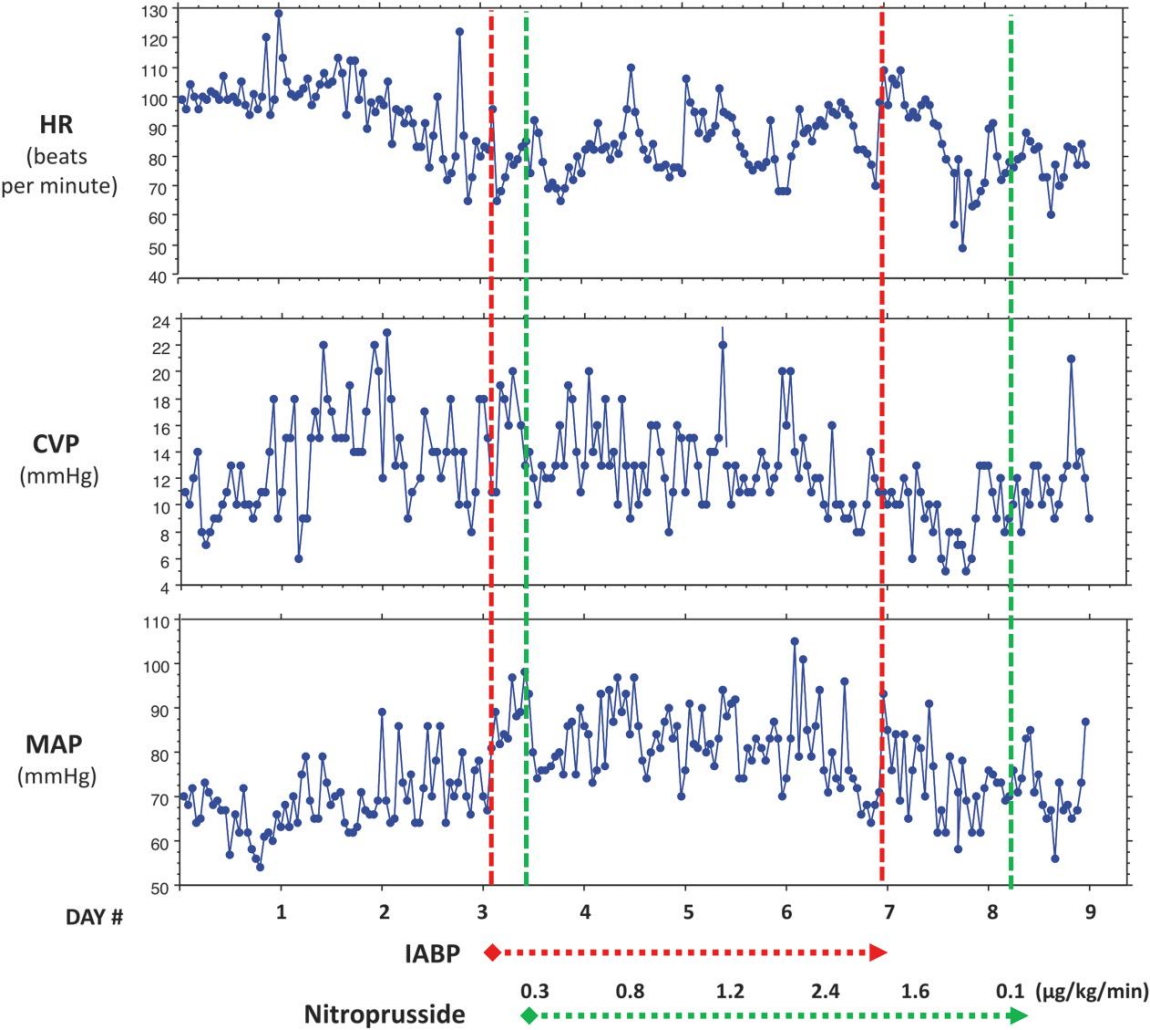
The temporal profiles of selected haemodynamic parameters: heart rate (HR), central venous pressure (CVP), and mean arterial pressure (MAP) according to days hospitalized at the tertiary care hospital. Dotted lines and arrows indicate the time from placement to removal of the intra-aortic balloon pump (IABP), and the time during which nitroprusside was administered, including dosage.

Due to cardiac dysfunction, noradrenaline was replaced with a dobutamine infusion, which was administered for the first 2 days. High-flow nasal cannula ventilatory support with nebulizers and respiratory physiotherapy was administered. Loop diuretics were given due to persistent pulmonary congestion. Levofloxacin and tazobactam/piperacillin were added, and the inflammatory parameters improved (*Figure 1*). Nonetheless, the patient appeared increasingly fatigued with limited respiratory reserve, mean arterial blood pressure (MAP) remained low, and heart rate was decreasing despite dobutamine infusion in adequate doses (*Figure 5*).

Treatment with IABP was initiated to offload the failing ventricle. Shortly after IABP placement, MAP rose, allowing the addition of nitroprusside in increasing doses to further reduce afterload. Mean arterial blood pressure remained higher than before IABP despite treatment with nitroprusside (*Figure 5*).

On the third day of IABP treatment, an ACE-inhibitor was introduced. Echocardiography shortly before weaning from IABP showed significant improvement in ventricular systolic function (see Supplementary material online, *Videos S3* and *S4*). Central venous oxygen saturation had increased from 67% to 77% (*Figure 1*), and the patient’s general condition had improved considerably. At the time of discharge from his local hospital 10 days later, he had made a full recovery.

## Discussion

The main haemodynamic effects of the IABP in patients with normal hearts have been described previously.^7,8^ When the balloon inflates in diastole, it displaces blood both forwards and backwards, whereby blood is redistributed to the coronary arteries and organ perfusion is augmented.^7^ Balloon deflation reduces end-diastolic aortic pressure and shortens the isovolumetric ventricular contraction. This decreases wall stress as well as myocardial oxygen demand, and potentially also reduces the regurgitant volume in the presence of atrioventricular valve insufficiency. Accordingly, IABP treatment is associated with increased coronary blood flow, increased cardiac output, and reduced afterload. Although IABP treatment is not standard of care in sepsis cardiomyopathy, there are reports of its successful use as circulatory support.^9^

Our hypothesis was that the haemodynamic decompensation resulted from pneumonia in addition to latent heart failure, and that IABP might increase cardiac output and decrease ventricular filling pressure. Unfortunately, and similar to previous case reports, echocardiographic or invasive data directly demonstrating such effects were not obtained. However, the reversal of pulmonary congestion and increased blood pressure were probably related to augmentation of cardiac output. Moreover, the increase in central venous oxygen saturation suggests an increase in cardiac output, according to Fick’s principle.

The increase in MAP allowed for treatment with nitroprusside to further reduce afterload, which probably also contributed to improved haemodynamics. Our observations indicate that IABP and nitroprusside were the main contributors to the patient’s recovery, but it is not possible to discern which factor that had the strongest impact, alongside anti-infective treatment.

The Fontan circulation is critically dependent on the transpulmonary gradient generated by the difference between central venous pressure and ventricular filling pressure. Accordingly, an IABP reducing ventricular filling pressure could be particularly useful in a Fontan patient with acute ventricular failure. This is in accordance with the observation that IABP after a Fontan procedure in children was effective only in patients with cardiac dysfunction.^4–6^ Possibly, haemodynamic monitoring, e.g. by measuring pulmonary capillary wedge pressure, might help in the selection of Fontan patients with acute heart failure who might benefit from IABP treatment. With respect to other modalities of circulatory support in Fontan patients, extracorporeal membrane oxygenation has been used as a short-term bridge to other treatment, albeit with higher rates of morbidity and mortality than in non-Fontan patients.^10^ Ventricular assist devices (VAD) are increasingly common amongst Fontan patients, particularly in those awaiting heart transplantation, whereas Impella support has only been described in a few case reports.^11^ Extracorporeal membrane oxygenation and VAD, both far more invasive than IABP, were not considered appropriate in our patient.

Prior to initiation of IABP treatment, the patient was hypotensive, and there was a drop in heart rate. Relative bradycardia is frequently seen in patients with septic shock,^12^ yet the clinical significance of this in patients with complex congenital heart disease is unclear. Moreover, chronotropic incompetence may be associated with myocardial ischaemia,^13^ and although ECG and troponin evidence were inconclusive, hypotension might induce ischaemia in our patient due to his potentially vulnerable coronary perfusion via the DKS anastomosis and hypoplastic aortic root.

Pneumonia may increase pulmonary vascular resistance through vasoconstriction induced by hypoxia and inflammatory vasocongestion. This may contribute to inadequate filling of the ventricle and reduce the IABP effect. Determining of the impact of these factors in our patient is difficult, but since his pneumonia affected parts of only one pulmonary lobe, the impact was probably not substantial.

## Conclusion

Treatment with IABP probably had a significant impact on recovery in our Fontan patient with acute pneumonia and heart failure. Although ‘hard’ data demonstrating the haemodynamic effects were not available, we hypothesize that IABP treatment may be particularly useful in the treatment of acute heart failure in Fontan patients due to their dependency on low ventricular filling pressure. This hypothesis should be tested by obtaining invasive measurements before and after IABP placement.

## Supplementary Material

ytae289_Supplementary_Data

## Data Availability

The data underlying this article are available in the article and in its online Supplementary material.

## Lead author biography

Miriam S. Langseth lives in Oslo and works as a physician in the Department of Cardiology at Oslo University Hospital, Rikshospitalet. She is a qualified General Internal Medicine specialist, and is currently working as a cardiology trainee/registrar. In 2022, she defended her PhD entitled ‘Neutrophil extracellular traps (NETs) in coronary artery disease. Prognostic value and roles in atherothrombosis’.
